# Impact of energy restriction during late gestation on the muscle and blood transcriptome of beef calves after preconditioning

**DOI:** 10.1186/s12864-018-5089-8

**Published:** 2018-09-25

**Authors:** Leticia P Sanglard, Moysés Nascimento, Philipe Moriel, Jeffrey Sommer, Melissa Ashwell, Matthew H Poore, Márcio de S Duarte, Nick V L Serão

**Affiliations:** 1 Department of Animal Science, Iowa State University, Ames, 50011 USA; 2 Department of Animal Science, North Carolina State University, Raleigh, 27695 USA; 3 Department of Statistics, Universidade Federal de Viçosa, Viçosa, 36570-000 Brazil; 4 Range Cattle Research and Education Center, University of Florida, Ona, Florida, 33865 USA; 5 Department of Animal Science, Universidade Federal de Viçosa, Viçosa, 36570-000 Brazil; 6 Instituto Nacional de Ciência e Tecnologia – Ciência Animal, Viçosa, 36570-000 Brazil

**Keywords:** RNA-seq, Fetal development, Fetal programming, Gene expression

## Abstract

**Background:**

Maternal nutrition has been highlighted as one of the main factors affecting intra-uterine environment. The increase in nutritional requirements by beef cows during late gestation can cause nutritional deficiency in the fetus and impact the fetal regulation of genes associated with myogenesis and immune response.

**Methods:**

Forty days before the expected calving date, cows were assigned to one of two diets: 100% (control) or 70% (restricted group) of the daily energy requirement. Muscle samples were collected from 12 heifers and 12 steers, and blood samples were collected from 12 steers. The objective of this work was to identify and to assess the biological relevance of differentially expressed genes (DEG) in the skeletal muscle and blood of beef calves born from cows that experienced [or not] a 30% energy restriction during the last 40 days of gestation.

**Results:**

A total of 160, 164, and 346 DEG (*q*-value< 0.05) were identified in the skeletal muscle for the effects of diet, sex, and diet-by-sex interaction, respectively. For blood, 452, 1392, and 155 DEG were identified for the effects of diet, time, and diet-by-time interaction, respectively. For skeletal muscle, results based on diet identified genes involved in muscle metabolism. In muscle, from the 10 most DEG down-regulated in the energy-restricted group (REST), we identified 5 genes associated with muscle metabolism and development: *SLCO3A1*, *ATP6V0D1*, *SLC2A1*, *GPC4*, and *RASD2*. In blood, among the 10 most DEG, we found genes related to response to stress up-regulated in the REST after weaning, such as *SOD3* and *INO80D*, and to immune response down-regulated in the REST after vaccination, such as *OASL, KLRF1*, and *LOC104968634.*

**Conclusion:**

In conclusion, maternal energy restriction during late gestation may limit the expression of genes in the muscle and increase expression in the blood of calves. In addition, enrichment analysis showed that a short-term maternal energy restriction during pregnancy affects the expression of genes related to energy metabolism and muscle contraction, and immunity and stress response in the blood. Therefore, alterations in the intra-uterine environment can modify prenatal development with lasting consequences to adult life.

**Electronic supplementary material:**

The online version of this article (10.1186/s12864-018-5089-8) contains supplementary material, which is available to authorized users.

## Background

Maternal nutrition has been highlighted as one of the main factors affecting the intra-uterine environment [1]. There is an increase in nutritional requirements of beef cows during late gestation [2], and if not met, the limited amount of nutrients available for optimum fetal development may affect prenatal physiological functions and, consequently, result in impaired post-natal growth and performance of the offspring [3, 4]. The last trimester of gestation is therefore a critical period for fetal growth [3].

Metabolizable energy restriction has been shown to down-regulate the mitogenic responses of T lymphocytes in beef calves [4, 5]. Prenatal programming of physiological systems can alter the growth and the function of organs and pathology into adulthood [6]. In other words, nutritional deficiency may impact the regulation of genes associated with myogenesis and immune response in the fetus, which can have long-term effects on progeny performance [3].

The deposition of skeletal muscle has significant implications to the animal meat-production systems. Muscle fibers are formed mainly during mid-gestation, and the definitive number of fibers is determined before birth [7]. On the other hand, fiber hypertrophy occurs during the late gestational period and continues following birth [7]. In addition, since a well-developed immune system protects the animal from pathogens and other stressors, healthy animals present better performance compared to compromised animals during growth and development [8]. Moreover, the prenatal programming of the immune system would give rise to changes that persist over the course of the animal’s life [9].

Transcriptome studies have shown changes in fetal growth and immune factors, especially during the final trimester of gestation. Studies have reported differentially expressed genes (DEG) in the muscle of the fetus as an effect of different energy sources in maternal diets, which ultimately change the metabolizable energy intake [9, 10]. For example, a decrease in expression of genes involved in muscle synthesis and differentiation, tissue and organ development, chromatin biology, and metabolic processes have been reported in calves as an effect of maternal diet during pregnancy based on corn compared with a diet based on alfalfa haylage, and dried corn distillers grains [10]. In addition, O’Loughlin et al. [11] identified DEG involved in cytokine signaling, transmembrane transport, hemostasis and G-protein-coupled receptor signaling as a response to weaning stress in calves.

The preconditioning phase is a critical period for beef calves, since animals pass through stressful procedures such as vaccination, weaning, and diet changes [12, 13]. As a response, there is a mobilization of protein from muscle [14] to increase the synthesis of proteins and cells involved in the immune system [15]. Consequently, the growth performance is compromised due to limited availability of nutrients to skeletal muscle development. Also during this period, there is a decrease in energy and protein intake [16] which may reduce the immune capacity of the animal and, consequently, impacts the vaccination response [13].

We hypothesized that maternal energy restriction would cause changes in the expression of genes related to muscle development and immune response in preconditioned beef calves born from cows that experienced [or not] a short-term energy restriction (30% of total energy requirements) during the last 40 d of gestation. Therefore, the objectives of this work were (1) to identify DEG in the muscle and blood of beef calves born from cows with and without energy restriction, (2) to assess the biological relevance of DEG, and (3) to investigate the relationships of DEG through gene networks.

## Methods

The animal trial was conducted at the Mountain Research Station (Waynesville, NC; 35.48° N, 82.99° W; elevation 659 m) at North Carolina State University, from January to November 2015, using animals from its research herd. The study was conducted in compliance with all welfare regulations, with all the study procedures being approved by the Institutional Animal Care and Use Committee of North Carolina State University (15–054-A). After the study, all animals returned to the research herd.

### Animals and diets

Thirty receptors multiparous, nonlactating, spring-calving pregnant Angus cows with an average body weight of 631 ± 15 kg, age of 5.2 ± 0.98 years, and body condition score of 6.3 ± 0.12 were used in this study. Cows were sired by two sires, and forty days before the expected calving date, the cows were randomly assigned to one of the two isonitrogenous diets: the control group (CTRL) and the restricted group (REST). Animals received total-mixed diets formulated to provide 100% (CTRL) or 70% (REST) of the daily net energy requirement for maintenance of a 630 kg beef cow at 8 months of gestation [17]. Animals were randomly assigned to pens according to the treatment (*n* = 5 pens/treatment). Immediately after calving, cow–calf pairs were transferred to 1 of 6 tall fescue pastures with free choice access to water and a complete mineral mix. Cows received dietary treatments for 40 ± 5.1 days. All male calves were castrated by banding immediately after birth. Cows and calves were managed as a single group and rotated among pastures monthly from calving until weaning (approximately 227 days of age). From weaning (day 0) until 40 days post weaning (dpw), calves were assigned to a preconditioning period. Additional detailed information regarding the nutritional information of the diets, feeding strategies, and the design of the study has been described in Moriel et al. [18] .

### Preconditioning

At weaning (0 dpw), calves were individually treated with doramectin for internal and external parasites (5 mL subcutaneous; Dectomax injectable; Zoetis Inc., Kalamazoo, MI). At 8 dpw, calves were vaccinated against infectious bovine rhinotracheitis virus, bovine viral diarrhea virus types 1a and 2, parainfuenza 3 virus, *Mannheimia haemolytica* (2 mL subcutaneous; Bovi Shield Gold One Shot; Zoetis Inc.), and Clostridium spp. (2 mL subcutaneous; Ultrabac 7; Zoetis Inc.). At 21 dpw, calves received 2 mL subcutaneous boosters of Bovi Shield Gold 5 (Zoetis Inc.) and Ultrabac 7. This vaccination protocol was used to replicate the standard protocol used by the local preconditioning alliance (Mountain Cattle Alliance, Canton, NC) [19, 20].

### Tissue collection

At 21 dpw, a biopsy of the skeletal muscle *Longissimus dorsi* was performed in all calves (12 steers and 12 heifers) at the level of the pelvis between the iliac, coxal and ischial tuberosity to obtain a minimum sample of tissue for subsequent gene expression analysis. The muscle biopsies were placed into a 2 mL Cryovial tube containing RNAlater (Ambion Inc., Austin, TX, USA) and stored in a − 20 °C freezer for subsequent laboratory analysis. In addition, the blood samples (10 mL) were collected from all steers via jugular venipuncture into Tempus Blood RNA Tubes (Life Technologies, Carlsbad, CA, USA) at 0, 3, 6, and 15 dpw for subsequent gene expression analysis. Blood samples were immediately put on ice and stored at − 80 °C until later laboratory analysis. Although 30 cows were initially used in the trial, subsequent analyses were performed on a subset of samples in order to reduce costs. Thus, a random sample of 24 calves (12 steers and 12 heifers) and 12 steers (one from each pen) were used for muscle and blood analyses, respectively. Since, we had four time points for blood, we opted to analyze just one sex to avoid increasing the complexity of the statistical models and the excessive costs.

### RNA extraction, sequencing, and bioinformatics

Total RNA from muscle and blood samples were extracted using RNeasy Fibrous Tissue Mini Kit (Qiagen Inc., Germantown, MD, USA) and Tempus™ RNA isolation kit (Applied Biosystems, Foster City, CA, USA), respectively. The RNA quantity and quality were determined by Agilent 2100 Bioanalyzer (Agilent Technologies, Inc., Santa Clara, CA, USA). One muscle and one blood sample were excluded from subsequent analysis due to low RNA integrity number (RIN) score (RIN < 8). All remaining samples were sent out to the Genomic Sciences Laboratory (North Carolina State University, Raleigh, NC, USA) for library construction and RNA-sequencing. Sequencing was performed on an Illumina NextSeq 500 instrument (Illumina, Inc., San Diego, USA), generating 75 bp paired-reads and 150 bp single-end reads for muscle and blood, respectively. The difference was to avoid excessive cost with the paired-end analysis since we had higher number of samples and it has been proposed that single-ends are sufficient to detect difference in expression in RNA-seq analysis. A total of 2 and 3 flow cells were used, respectively, for muscle and blood samples, with approximate equal representation of treatments between flow cells.

The quality of raw reads were evaluated with FastQC [21]. Sequence reads for each sample were mapped to *Bos taurus* UMD3.1 reference genome using Bowtie2 [22]. The number of counts for each sample was obtained with the *Subread* package from SourceForge [23]. A total of 2,076,680,240 paired-ends and 1,353,110,288 single-ends reads were generated for muscle and blood samples, respectively, with an average of 90,290,445 and 28,789,580 reads/sample, respectively. Reads were mapped to a total of 19,045 genes (77.43%) and 19,331 genes (78.59%) for muscle and blood, respectively. This was from a total of 24,596 genes annotated in the *Bos taurus* reference genome UMD3.1. Genes with a total of counts less than four times the number of samples were eliminated to avoid low counts across multiple samples, resulting in a final set of 15,255 and 13,512 genes for muscle and blood, respectively. For normalization of the data, we used the Trimmed Mean of M-values (TMM) to calculate the normalized factors (*TCC* package [24]). The normalized library size was obtained by dividing the total library size by the normalized factors.

### Statistical analyses

Gene expression data was analyzed with a negative binomial model with a log link function [25]. Different models were tested for each tissue. For muscle, a negative binomial function was used in the following model:

$$ {Y}_{ij klmn}=\mu +{D}_i+{S}_j+{\left(D\ast S\right)}_{ij}+{B}_k+{P}_l+{\upbeta}_1\left({R}_{ij klmn}-\overline{R}\right)+{\upbeta}_2\left({DOT}_{ij klmn}-\overline{DOT}\right)+{SS}_n+\mathit{\log}\left({L}_{ij klmn}\right)+{\varepsilon}_{ij klmn} $$

where, *Y*_*ijklmn*_ is the raw number of counts; *μ* is the intercept, *D*_*i*_ is the fixed-effect of the i^th^ Diet; *S*_*j*_ is the fixed effect of the j^th^ Sex; *B*_*k*_ is the fixed effect of the k^th^ Batch (sequencing lane); *P*_*l*_ is the fixed effect of the l^th^ Pen; *β*_*1*_ is the partial regression coefficient for the covariate RIN scores (*R*); *β*_*2*_ is the partial regression coefficient for the covariate Days on Dietary Treatment (DOT); *SS*_*n*_ is the fixed effect of the n^th^ Service Sire; *L*_*ijklmn*_ is the TMM-normalized library size, used as an offset; and *Ɛ* is the random residual associated with *Y*_*ijlkmn*_. In addition to this model, 3 other reduced models were tested, in which the effects of pen and/or the interaction were removed. All models were used for each of the 15,255 genes, and the final model for each gene was chosen based on Akaike information criterion (AIC).

For the gene expression analysis of blood samples, a negative binomial function was used in the following model:

$$ {Y}_{ij klmn}=\mu +{D}_i+{T}_j+{\left(D\ast T\right)}_{ij}+{B}_k+{\upbeta}_1\left({R}_{ij klmn}-\overline{R}\right)+{\upbeta}_2\left({DOT}_{ij klmn}-\overline{DOT}\right)+{SS}_n+\mathit{\log}\left({L}_{ij klmn}\right)+{\varepsilon}_{ij klmn} $$

where, *Y*_*ijklmn*_, *μ*, *D*, *B*_*k*_, β_1_, *R*_*ijklmn*_, *DOT*_*ijklmn*_, *SS*_*n*_*, L*_*ijklmn*_ and *Ɛ*_*ijklmn*_ are as previously defined. *T*_*j*_ is the fixed effect of the j^th^ Time. A reduced model without interaction was tested, and analyses were performed using three covariance structures for the residuals: first-order autoregressive, compound symmetry, and independent residuals. Therefore, 6 models were tested for each of the 13,512 genes, and the final model for each gene was selected based on AIC. Satterthwaite approximation to account for sample variances was used to determine the denominator degrees of freedom (df). The dispersion parameter of the model was calculated for each gene squeezing towards a global dispersion with an approach assuming mean and variance related by *σ*^2^ = *μ* + *aμ*^2^ [26], where *a* is a proportiallity constant, using the package e*dgeR* in R software [27].

Additional contrasts were constructed in order to answer biologically relevant questions in the analysis of blood samples. Contrasts were developed for both the interaction effect (diet-by-time) and the main effect of time. For the main effect of time, two contrasts with 1-df were constructed: (1) to test the effect of vaccination (i.e. average of days 0, 3, and 6, versus day 15) and (2) to evaluate the acute response to weaning (i.e. day 0 versus average of days 3 and 6). For the interaction effect, these same contrasts were constructed, but accommodating their interaction with diet.

False-discovery rate (FDR [28]) was used to adjust the *P-*values (*q*-values) of model terms due to multiple testing. Significant DEG were identified at *q*-value ≤0.05 for all analyses. All data were analyzed using the GLIMMIX procedure of SAS 9.4 (Statistical Analysis System Institute, Inc., Cary, NC, USA).

### Functional annotation analysis

The enrichment of Gene Ontology (GO) terms associated with DEG was analyzed using PANTHER Enrichment Analysis [29]. Different DEG lists were created based on the significance (*q*-value < 0.05) of effects in the model. For the muscle data, analyses were performed separately for DEG based on diet and sex. For blood, we performed 3 analyses, one based on the effect of diet, and two based on the previously described contrasts (effect of vaccination and weaning). The *Bos taurus* genome was used as the background list. Biological Processes was considered significant at *P*-value < 0.05.

### Gene-network analysis

Genes networks were constructed for the effect of diet including all DEG in muscle and all DEG in muscle and blood. Then, due to the difficult visualization in the previous networks, we have created more two gene networks including the 20 most DEG in the muscle and the 10 most DEG for the effect of diet in muscle associated with the 10 most DEG in blood. Partial correlation networks were constructed using gene counts pre-adjusted for all effects in the model, with the exception of diet (the effect of interest). In other words, the data used in this analysis, for each individual, represented the sum of the estimated diet effect and its residual. For the network including DEG from muscle and blood tissues, only data from steers were used as only male offspring had data using both tissues. For the gene networks including all DEG, connections between genes (i.e. nodes) were included when a pair of genes showed a partial correlation greater than |0.8|. The correlation matrix and the gene-networks were constructed using the *ppcor* [30] and *qgraph* [31] packages in R software [27].

## Results and discussion

Alterations in the intra-uterine environment, such as those caused by maternal nutrition during pregnancy, can modify prenatal development, which can lead to positive or negative consequences to the adult life of the animal [2, 32]. In addition to the prenatal susceptibility, the pre-conditioning phase is also critical for offspring development since the animal is going through a stressful period and may have its performance compromised. Therefore, a well-developed immune system, associated with proper development of muscle tissue, would favor the productive performance of the animal. Our study focused on the effect of maternal energy restriction during late gestation on the transcriptomic profile in the skeletal muscle and blood tissues in the offspring.

### DEG identification

**Fig. 1:**
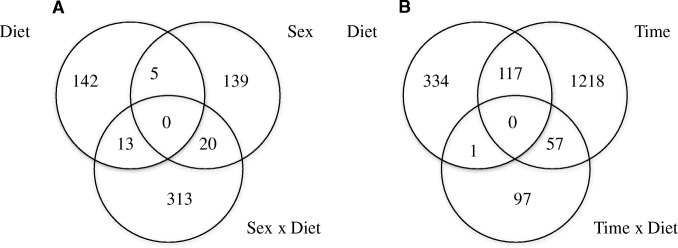
Venn Diagram with the number of differentially expressed genes (DEG; *q*-value < 0.05) for the effects of diet, sex, and diet-by-sex for muscle (**a**), and diet, sex and diet-by-time for blood (**b**)

The number of DEG identified in this study is shown in Fig. 1. A total of 160, 164, and 346 DEG (*q*-value < 0.05) were identified in the muscle for the effects of diet, sex, and diet-by-sex interaction, respectively (Fig. 1a). For blood, 452, 1392, and 155 DEG (*q*-value < 0.05) were identified for the effects of diet, time, and diet-by-time interaction, respectively (Fig. 1b). For the contrasts in blood tissue, there were 101 and 47 DEG (*P*-value < 0.05) for Weaning and Vaccination, respectively, based on the diet-by-time interaction effect (*q*-value < 0.05). For the main effect of Time (*q*-value < 0.05), there were 893 and 473 DEG (*P*-value < 0.05) for the Weaning and Vaccination contrasts, respectively.

**Fig. 2:**
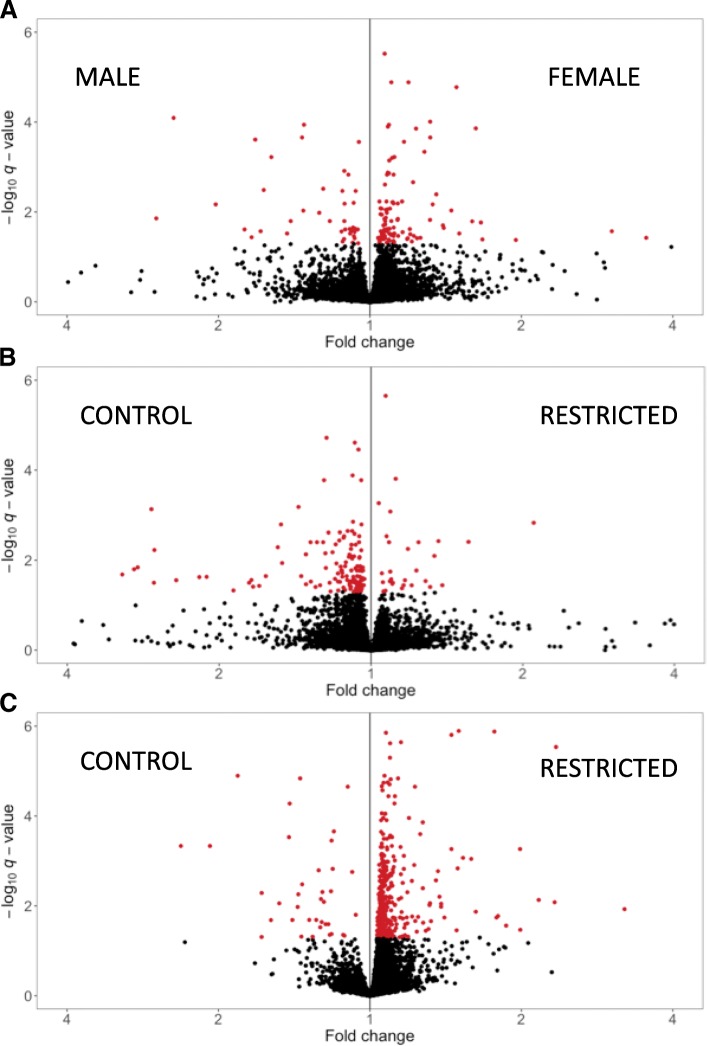
Volcano plots for the effect of sex (**a**) and diet (**b**) in the muscle tissue, and for the effect of diet (**c**) in the blood tissue. The Y-axis shows the –log10 *q*-values for the effects of sex (**a**) or diet (**b** and **c**), whereas the X-axis represents the fold change (FC), with FC equal 1 representing equal expression between the two compared groups. In A, FC values to the left and right represent the up-regulation of DEG in male and female calves, respectively. In B and C, FC values to the left and right represent up-regulation of DEG in the control and restricted diets, respectively. Genes with significant (*q*-value < 0.05) effect of diet are highlighted in red. Extreme values of FC (< or > 5) and *q*-value (< 1.0E-06) were removed from the plot

The volcano plots for the effect of diet are shown in Fig. 2. For the effect of sex in the muscle, there was a greater number of up-regulated genes in the females (117) compared with males (47; Fig. 2a). For the effect of diet, there was a greater number of down-regulated (131) genes in the REST compared to up-regulated (29) in the muscle tissue (Fig. 2b), suggesting that maternal energy restriction during late gestation may limit the expression of genes in the muscle of calves. In contrast, there was a much greater number of up-regulated genes in the REST (410) compared to down-regulated (42) in the blood (Fig. 2c). Although we do not have the data to conclude on this, this clear gene-regulation bias towards one diet or the other could be due to maternal energy restriction causing epigenetic modifications in the fetal genome.

### Enrichment analyses in the muscle

The results of the enrichment analysis for diet and sex are presented in Table 1. Results based on diet (*P* < 0.05) showed genes involved in the metabolisms of energy and muscle, i.e. *positive regulation of actin cytoskeleton reorganization* (GO:2000251)*, positive regulation of collagen biosynthetic process* (GO:0032967), *adipose tissue development* (GO:0060612) and *positive regulation of collagen metabolic process* (GO:0010714); and nervous system metabolism, i.e. *regulation of neuron differentiation* (GO:0045664), *regulation of neurogenesis* (GO:0050767), *regulation of nervous system development* (GO:0051960), and *negative regulation of neuron projection development* (GO:0010977). Enrichment analyses for the effect of sex showed that the DEG were enriched for categories related to acid nucleic metabolic process, such as *histone H3-K27 demethylation* (GO:0071557), *DNA-dependent DNA replication* (GO:0006261), *DNA replication initiation* (GO:0006270), and *DNA repair* (GO:0006281); and metabolism of carbohydrate and muscle, such as *positive regulation of glycoprotein biosynthetic process* (GO:0010560), *regulation of skeletal muscle contraction* (GO:0014819), and *regulation of glycogen metabolic process* (GO:0070873). These functional terms are related to chromatin biology and to epigenetics suggesting that these variations are responsible for the changes in the gene expression observed in the fetal tissues. Our study supports the hypothesis that males and females have different gene expression pattern during fetal development. The differences in offspring outcomes according to their sex as an effect of fetal programming has been reported in sheep [33, 34] and cows [35, 36]. In this study however, we did not look at the effect of interaction diet-by-sex since, in the muscle analysis, few genes were selected for the model containing interaction. Additional results (*P* < 0.05) including biological function GO terms for the effects of diet and sex can be seen in Additional file 1.

**Table 1:** Enrichment analysis showing 20 most overrepresented^1^ Biological Process for differentially expressed genes^2^ for the effects of diet and sex in the muscle tissue

Biological Process	# genes	FE	*P*-value
*Diet*			
Positive regulation of actin cytoskeleton reorganization (GO:2000251)	2	21.68	4.88E-03
Peptidyl-proline hydroxylation (GO:0019511)	2	21.68	4.88E-03
Positive regulation of blood vessel endothelial cell migration (GO:0043536)	3	13.21	1.90E-03
Negative regulation of myeloid cell differentiation (GO:0045638)	4	9.56	1.02E-03
Membrane depolarization (GO:0051899)	3	9.4	4.70E-03
Regulation of protein targeting (GO:1903533)	4	6.33	4.26E-03
Negative regulation of hemopoiesis (GO:1903707)	4	5.75	5.90E-03
Regulation of cell morphogenesis involved in differentiation (GO:0010769)	6	4.55	2.44E-03
Regulation of mitochondrion organization (GO:0010821)	5	4.49	5.87E-03
Regulation of neuron projection development (GO:0010975)	8	4.27	7.09E-04
Regulation of intracellular transport (GO:0032386)	7	3.55	4.16E-03
Regulation of cell morphogenesis (GO:0022604)	8	3.33	3.28E-03
Regulation of neuron differentiation (GO:0045664)	9	3.32	1.86E-03
Positive regulation of transport (GO:0051050)	14	2.92	3.81E-04
Regulation of plasma membrane bounded cell projection organization (GO:0120035)	8	2.86	7.79E-03
Regulation of neurogenesis (GO:0050767)	9	2.68	7.28E-03
Regulation of nervous system development (GO:0051960)	10	2.55	6.61E-03
Regulation of transport (GO:0051049)	20	2.08	2.13E-03
Cell surface receptor signaling pathway (GO:0007166)	20	1.87	6.52E-03
Regulation of localization (GO:0032879)	25	1.74	7.58E-03
*Sex*			
Histone H3-K27 demethylation (GO:0071557)	2	55.66	1.04E-03
Kinetochore assembly (GO:0051382)	3	52.18	5.64E-05
Positive regulation of sodium ion transmembrane transporter activity (GO:2000651)	2	39.76	1.76E-03
Kinetochore organization (GO:0051383)	3	34.79	1.52E-04
Positive regulation of glycoprotein biosynthetic process (GO:0010560)	3	34.79	1.52E-04
Regulation of skeletal muscle contraction (GO:0014819)	2	34.79	2.19E-03
Positive regulation of glycoprotein metabolic process (GO:1903020)	3	29.82	2.25E-04
Centromere complex assembly (GO:0034508)	3	21.97	4.97E-04
Establishment of melanosome localization (GO:0032401)	3	20.87	5.68E-04
Establishment of pigment granule localization (GO:0051905)	3	19.88	6.46E-04
Melanosome localization (GO:0032400)	3	19.88	6.46E-04
Pigment granule localization (GO:0051875)	3	18.98	7.30E-04
ATP hydrolysis coupled transmembrane transport (GO:0090662)	3	16.06	1.14E-03
ATP hydrolysis coupled proton transport (GO:0015991)	3	16.06	1.14E-03
Energy coupled proton transmembrane transport, against electrochemical gradient (GO:0015988)	3	14.91	1.38E-03
Regulation of glucose metabolic process (GO:0010906)	5	9.53	2.41E-04
Regulation of cellular carbohydrate metabolic process (GO:0010675)	5	7.65	6.29E-04
Regulation of carbohydrate metabolic process (GO:0006109)	5	6.5	1.26E-03
Chromosome organization (GO:0051276)	17	2.59	3.56E-04
Cellular macromolecular complex assembly (GO:0034622)	14	2.57	1.32E-03

^1^*P*-value < 0.05;

^2^*q*-value < 0.05;

FE = fold enrichment

### Major DEG in the muscle

The 10 most significant up- and down-regulated DEG in REST are summarized in Table 2. Of these, the most significant down- and up-regulated genes in the REST were *SLCO3A1* and *ETNPPL*, with a fold change (FC) [95% confidence interval] of 0.77 [0.71, 0.84] (*q*-value = 4.58E-07) and 1.32 [1.18, 1.48] (*q*-value = 1.56E-04), respectively. The most extreme FC was observed for *SPATA1*, with 2.28 [1.54, 3.39] (*q*-value = 3.91E-03). From the 10 most down-regulated genes in REST, we identified 5 genes associated with muscle metabolism and development as well as with energy metabolism: (1) *SLCO3A1*, a membrane transporter of the thyroid hormone involved in satellite cell differentiation [37]; (2) *ATP6V0D1*, involved in oxidative phosphorylation [38]; (3) *SLC2A1*, a facilitator of glucose transport; (4) *GPC4*, involved in cell proliferation [39]; and (5) *RASD2*, expressed in satellite cells and involved in cellular movement and cell cycle. This connection of major genes with energy metabolism associated with the relevant functional terms in the enrichment analysis may indicate a shift in the way the tissues are generating energy in the REST compared with CRTL.

**Table 2:** The 10 most significant up- and down-regulated differentially expressed genes^1^ in the muscle tissue for the effects of Diet^2^ and Sex^3^

Gene symbol	Gene name	Biological function	FC [CI]	*q*-value
*DEG for effect of diet*				
SPATA1	Spermatogenesis associated 1	Involved in male fertility [58]	2.28 [1.54, 3.39]	3.91E-03
SYT3	Synaptotagmin 3	Involved in glucose metabolism [50]	1.89 [1.39, 2.57]	3.75E-03
FZD5	Frizzled class receptor 5	Wnt/Wingless (Wg) signals [52]	1.84 [1.35, 2.5]	8.00E-03
AQP4	Aquaporin 4	Water channels [59]	1.62 [1.28, 2.06]	3.97E-03
CHN1	Chimerin 1	GTPase-activating protein [60]	1.48 [1.22, 1.82]	5.59E-03
ETNPPL	Ethanolamine-phosphate phospholyase	Associated to fatty acid and lipid metabolic process [49]	1.33 [1.19, 1.48]	1.56E-04
SMIM11A	Small integral membrane protein 11A		1.26 [1.14, 1.39]	8.36E-04
ETFRF1	Electron transfer flavoprotein regulatory factor 1	Involved in fatty acid oxidation [51]	1.24 [1.12, 1.37]	3.97E-03
TMCC2	Transmembrane and coiled-coil domain family 2	Play a role in neurodegeneration [61]	1.21 [1.1, 1.31]	2.91E-03
TRAPPC6B	Trafficking protein particle complex 6B	Vesicle transport [62]	1.1 [1.06, 1.15]	5.42E-04
ATP6V0D1	ATPase H+ transporting V0 subunit d1	Involved in oxidative phosphorylation [43]	0.89 [0.85, 0.93]	1.67E-04
RTF2	Replication termination factor 2	Riboflavin transporters [63]	0.89 [0.84, 0.94]	1.62E-03
MLX	MAX dimerization protein	Control cell cycle [64]	0.82 [0.77, 0.88]	2.44E-05
GPC4	Glypican 4	Involved in cell proliferation [44]	0.81 [0.74, 0.89]	1.41E-03
TSPAN7	Tetraspanin 7	Maturation of glutamatergic synapses [65]	0.81 [0.74, 0.88]	1.31E-04
SLCO3A1	Solute carrier organic anion transporter family member 3A1	Thyroid hormone membrane transporter involved in satellite cell differentiation [42]	0.77 [0.71, 0.84]	4.58E-07
RASD2	RASD family member 2	Involved in cellular movement and cell cycle [48]	0.76 [0.68, 0.86]	2.25E-03
LAT	Inker for activation of T cells	T cell activation [66]	0.63 [0.53, 0.75]	1.91E-05
SLC2A1	Solute carrier family 2 member 1	Glucose transporter [67]	0.62 [0.51, 0.75]	1.67E-04
RASL10B	RAS like family 10 member B	Potential tumor suppression [68]	0.51 [0.38, 0.68]	6.60E-04
*DEG for effect of sex*				
FAAP24	Fanconi anemia core complex associated protein 24	DNA damage response [69]	2.14 [1.6, 2.87]	1.67E-05
TXLNG	Taxilin gamma		1.93 [1.59, 2.35]	8.48E-10
KDM6A	Lysine demethylase 6A	Demethylase [70]	1.67 [1.51, 1.85]	< 1.00E-13
ZRSR2	Zinc finger CCCH-type, RNA binding motif and serine/arginine rich 2		1.61 [1.39, 1.88]	4.27E-08
EIF2S3	Eukaryotic translation initiation factor 2 subunit gamma	Play a role in pituitary development and insulin secretion [71]	1.48 [1.38, 1.6]	< 1.00E-13
SRD5A3	Steroid 5 alpha-reductase 3	Produces steroid hormones [72]	1.38 [1.24, 1.53]	6.49E-08
TMCC2	Transmembrane and coiled-coil domain family 2		1.28 [1.16, 1.4]	1.30E-05
SYAP1	Synapse associated protein 1		1.27 [1.21, 1.34]	< 1.00E-13
USP9X	Ubiquitin specific peptidase 9, X-linked	TGF-β signaling [53]	1.25 [1.14, 1.37]	1.15E-04
ICMT	Isoprenylcysteine carboxyl methyltransferase	Methyltransferase [73]	1.24 [1.13, 1.35]	1.26E-04
ARID1B	AT-rich interaction domain 1B	Regulation of cell cycle [74]	0.87 [0.82, 0.93]	2.77E-04
ZBED1	Zinc finger BED-type containing 1	Associated with cell proliferation [75]	0.75 [0.66, 0.85]	1.22E-03
ADAM1B	A disintegrin and metallopeptidase domain 1b	Involved in male fertilization [76]	0.54 [0.41, 0.69]	1.15E-04
ANOS1	Anosmin 1	Cell adhesion [77]	0.53 [0.4, 0.69]	2.20E-04
GCGR	Glucagon receptor	Glucagon receptor [78]	0.44 [0.3, 0.63]	6.00E-04
ACTL8	Actin like 8		0.4 [0.27, 0.59]	2.45E-04
CD3G	CD3g molecule	T-cell receptor [79]	0.28 [0.17, 0.46]	8.08E-05
RIMS1	Regulating synaptic membrane exocytosis 1	Maintenance of normal synaptic function [80]	0.14 [0.1, 0.19]	< 1.00E-13
LOC107131205	Lysine-specific demethylase 6A-like	Antioxidant [81]	0.07 [0.06, 0.08]	< 1.00E-13
LOC107131189	Eukaryotic translation initiation factor 2 subunit 3, Y-linked-like		0.01 [0.01, 0.01]	< 1.00E-13

^1^*q*-value < 0.05;

^2^Up- and down-regulated in the restricted group compared with the control are represented by values bigger and smaller than 1, respectively;

^3^Up- and down-regulated in females compared with males are represented by values bigger or smaller than 1, respectively;

Biological Function, based on cited reference;

FC = Fold change;

CI = 95% Confidence Interval

We propose that energy is being produced primarily through the glycolytic pathway instead of the oxidative pathway, which is less efficient and occurs as a result of lack of sufficient energy. Concordantly, Daniel et al. [40] showed that maternal dietary restriction during mid-gestation increases the number of fast (type II) muscle fibers, which generate energy through glycolytic pathways, as a compensatory process due to limited energy availability for muscle development. Mitochondrial oxidative phosphorylation is the primary pathway to produce energy for metabolic activities, which generates more adenosine triphosphate than glycolysis [41]; however, lack of energy can decrease the number of fast fibers [40], reducing the efficiency of energy production and, consequently, of metabolic activities. In accordance, Byrne et al. [42] suggested that caloric restriction may increase metabolism of amino acids to glucose via gluconeogenesis after finding increased gene expression levels associated with energy metabolism. Moreover, Peñagaricano et al. [9] showed that the maternal source of energy may affect gene expression in the muscle of sheep. These authors observed that dams fed a corn-based diet had greater expression of genes associated with embryonic and fetal development, skeletal muscle, tissue differentiation, muscle myosin complex and sarcomere organization, than dams fed alfalfa haylage and distillers grains. These results indicate that the energy-restricted diet may be impairing the development of muscle tissue as well as altering the energy metabolism in the offspring. In addition, Yang et al. [39] reported the gene *GP4* being less expressed due to hypermethylation in Chinese pigs, which are known for having less lean muscle mass and low growth rate, suggesting that the low expression may be occurring due to epigenetic modifications. The down-regulation of *RASD2* in the REST may also indicate that the maternal energy restriction alters the proliferation capacity of the satellite cells, controlling the skeletal muscle hypertrophy. Raja et al. [43] have shown that energy restriction during gestation alters temporal expression of myogenic regulatory factors in satellite cells impairing the fusion of cells isolated from 3-month old lambs. Therefore, alterations in maternal nutrition during pregnancy may lead to negative effects on postnatal myogenesis.

In contrast, the REST showed a higher expression of genes related to fatty acid and glucose metabolism, such as *ETNPPL*, associated to fatty acid and to lipid metabolic process [44]; *SYT3*, involved in glucose metabolism [45]; and *ETFRF1*, involved in fatty acid oxidation [46]. *SYT3* has been shown to be up-regulated in a mutated mice presenting a lean phenotype, resistance to high fat diet, and insulin resistance [45]. This finding suggests that the REST presents an impaired glucose metabolism compared with the CTRL group, which may be influencing the increase in expression of genes associated with fatty acid metabolism (*ETNPPL* and *ETFRF1).* In addition, the overexpression of *FZD5* in the REST may indicate an disruption on Wnt signaling, which is related to diverse functions in developmental processes, including glucose metabolism [47]. These findings suggest that energy restriction during late pregnancy affects glucose metabolism and enhances fatty acid metabolism.

The 10 most significant DEG for the effect of sex are summarized in Table 2. The most significant up-regulated gene in males was *RIMS1* with a FC of 0.14 [0.1, 0.19] (*q*-value = < 1.00E-13), and in females was *SYAP1* with a FC of 1.27 [1.21, 1.34] (*q*-value = < 1.00E-13). The most extreme FC was observed for *LOC107131189* with 0.01 [0.01, 0.01] (*q*-value = < 1.00E-13). From the 10 most DEG for the effect of sex, in accordance with the enrichment analyses, we identified genes associated with glucose metabolism, such as *GCGR* (up-regulated in males) and *EIF2S3* (up-regulated in females) and chromatin biology and epigenetic modifications, such as *KDM6A* and *ICMT* up-regulated in females compared with males. In accordance, glucose metabolism had been thought to differ between male and female embryos [48]. In addition, Alvarez et al. [49] found higher transcriptional expression in females compared with males. We propose that the X-linked gene inactivation in females may occur as a result of an imprinting mechanism leading to a total or partial maternal allele transcriptional repression [49], which can generate an up-regulation of genes in females compared with males (Fig. 2). These results suggest that the different gene expression levels occurs due to difference in sex chromosome dosage. The complete list of DEG (*q*-value < 0.05) for the effects of diet, sex, and interaction diet-by-sex is provided in Additional file 2.

### Enrichment analysis in the blood

The enrichment analyses for the different sets of DEG in the blood are presented in Table 3. Results based on diet showed general metabolic functions (*P* < 0.05), such as t*ranslation* (GO:0006412), *rRNA metabolic process* (GO:0016072), and *biosynthetic process* (GO:0009058) in addition to carbohydrate metabolism such as *regulation of carbohydrate metabolic process* (GO:0006109) and *glycogen metabolic process* (GO:0005977). In contrast, for results based on biologically relevant contrasts, there was an overrepresentation of biological processes related to immune response for the effect of vaccination, such as *immune system process* (GO:0002376), *regulation of response to stimulus* (GO:0048583), and *positive regulation of response to stimulus* (GO:0048584), in addition to other general biological processes, such as *localization* (GO:0051179), and *transport* (GO:0006810). For the effect of weaning, we found GO terms associated with response to stress and immune system, i.e. *cellular response to DNA damage stimulus* (GO:0006974)*, response to stress* (GO:0006950)*, regulation of lymphocyte activation* (GO:0051249) and *response to interleukin-4* (GO:0070670). These include *cellular response to DNA damage stimulus* (GO:0006974)*, response to stress* (GO:0006950)*, regulation of lymphocyte activation* (GO:0051249), *response to interleukin-4* (GO:0070670) and *immune system process* (GO:0002376), suggesting that the maternal nutrition during pregnancy influenced the response of the offspring to the immune challenge after weaning and vaccination. It has been shown that the immune challenge may elicit an acute phase response which decreases feed intake and increases protein demand to support the immune system [12, 13] leading to compromised growth. The energy-restricted diet may be impairing the animal’s response in this phase, since the energy metabolism might be compromised and less efficient for synthesizing protein for the immune system. Additional enrichment analysis results (*P* < 0.05) including under and overrepresented molecular function GO terms for the effects of diet and time can be seen in Additional file 3.

**Table 3:** Enrichment analysis showing 20 most overrepresented^1^ Biological Process for differentially expressed genes^2^ for diet and biologically relevant contrasts in the blood tissue

Biological Process	# genes	FE	*P*-value
*Diet*			
Regulation of carbohydrate metabolic process (GO:0006109)	3	6.54	1.37E-02
Translation (GO:0006412)	22	4.3	3.42E-08
Glycogen metabolic process (GO:0005977)	3	4.03	4.38E-02
Transcription initiation from RNA polymerase II promoter (GO:0006367)	4	3.81	2.47E-02
rRNA metabolic process (GO:0016072)	10	3.77	5.20E-04
Protein targeting (GO:0006605)	8	2.3	2.73E-02
Cellular component biogenesis (GO:0044085)	36	2.08	6.01E-05
Protein localization (GO:0008104)	18	1.72	2.71E-02
Organelle organization (GO:0006996)	39	1.51	1.36E-02
Biosynthetic process (GO:0009058)	55	1.49	4.07E-03
Cellular component organization or biogenesis (GO:0071840)	65	1.48	1.68E-03
Cellular component organization (GO:0016043)	54	1.32	3.77E-02
Primary metabolic process (GO:0044238)	119	1.19	3.26E-02
*Weaning*			
Translation (GO:0006412)	101	5.84	6.58E-42
Peptide biosynthetic process (GO:0043043)	101	5.64	8.81E-41
Amide biosynthetic process (GO:0043604)	103	5.09	3.67E-38
Peptide metabolic process (GO:0006518)	103	4.8	3.40E-36
Cellular amide metabolic process (GO:0043603)	106	4.08	7.70E-32
Ribosome biogenesis (GO:0042254)	37	3.65	1.55E-10
Organonitrogen compound biosynthetic process (GO:1901566)	125	2.68	4.22E-22
Cellular macromolecule biosynthetic process (GO:0034645)	181	2.21	4.73E-23
Macromolecule biosynthetic process (GO:0009059)	181	2.17	1.99E-22
Cellular nitrogen compound biosynthetic process (GO:0044271)	165	2.11	4.11E-19
Gene expression (GO:0010467)	168	1.85	3.42E-14
Cellular protein metabolic process (GO:0044267)	213	1.84	3.63E-18
Organic substance biosynthetic process (GO:1901576)	203	1.8	2.37E-16
Cellular biosynthetic process (GO:0044249)	198	1.8	9.94E-16
Biosynthetic process (GO:0009058)	204	1.76	1.74E-15
Organelle organization (GO:0006996)	178	1.74	6.55E-13
Cellular nitrogen compound metabolic process (GO:0034641)	232	1.7	3.41E-16
Protein metabolic process (GO:0019538)	232	1.64	1.84E-14
Cellular macromolecule metabolic process (GO:0044260)	293	1.62	1.85E-18
Cellular component organization or biogenesis (GO:0071840)	252	1.52	2.74E-12
Macromolecule metabolic process (GO:0043170)	320	1.42	2.33E-12
*Vaccination*			
Myeloid leukocyte activation (GO:0002274)	8	5.47	1.93E-04
Transition metal ion transport (GO:0000041)	9	5.07	1.32E-04
Positive regulation of response to external stimulus (GO:0032103)	14	3.22	2.06E-04
Positive regulation of GTPase activity (GO:0043547)	22	3.18	4.20E-06
Regulation of GTPase activity (GO:0043087)	23	2.87	1.27E-05
Positive regulation of hydrolase activity (GO:0051345)	28	2.4	3.93E-05
Regulation of response to external stimulus (GO:0032101)	26	2.35	9.76E-05
Cellular homeostasis (GO:0019725)	28	2.24	1.36E-04
Immune system process (GO:0002376)	58	1.93	2.65E-06
Regulation of hydrolase activity (GO:0051336)	40	1.92	1.71E-04
Positive regulation of response to stimulus (GO:0048584)	56	1.78	3.82E-05
Cellular response to organic substance (GO:0071310)	55	1.77	5.19E-05
Cellular response to chemical stimulus (GO:0070887)	67	1.75	1.25E-05
Localization (GO:0051179)	141	1.61	3.46E-09
Establishment of localization (GO:0051234)	110	1.6	6.83E-07
Transport (GO:0006810)	104	1.57	3.50E-06
Regulation of response to stimulus (GO:0048583)	97	1.54	2.14E-05
Cell differentiation (GO:0030154)	84	1.53	8.59E-05
Regulation of signal transduction (GO:0009966)	77	1.53	2.22E-04
Regulation of signaling (GO:0023051)	83	1.5	2.22E-04
Developmental process (GO:0032502)	118	1.39	1.65E-04

^1^*P*-value < 0.05;

^2^*q*-value < 0.05;

FE = fold enrichment

### Major DEG in blood tissue

**Fig. 3:**
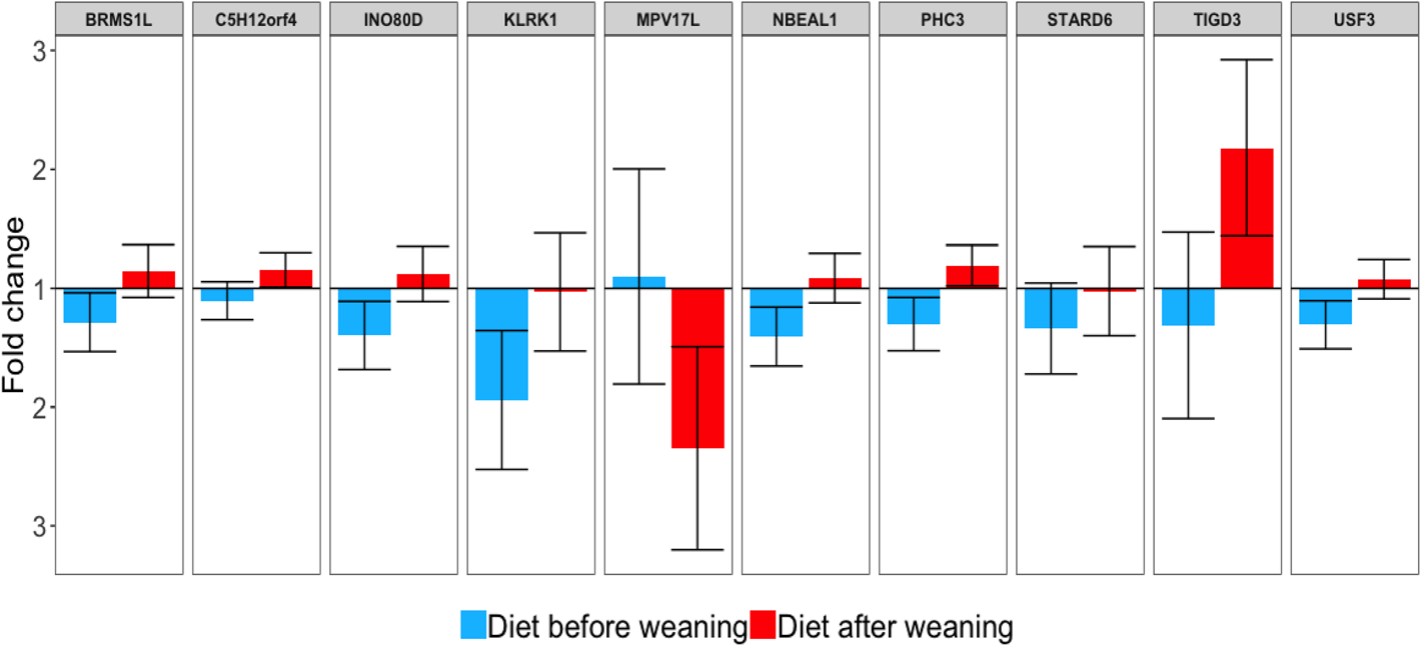
The 10 most differentially expressed genes (*q*-value < 0.05) showing interaction between the effects of diet and weaning in the blood tissue. Bars represent the fold change (FC) for the effect (*P*-value < 0.05) of diet before (blue) and after (red) weaning, with error bars representing the 95% confidence interval. Fold change values equal to 1 represent same expression between the diets (control and restricted). Fold change values on the top and bottom halves represent up-regulated DEG in the restricted and control groups, respectively. The name and function of these genes are summarized on Table 4

**Fig. 4:**
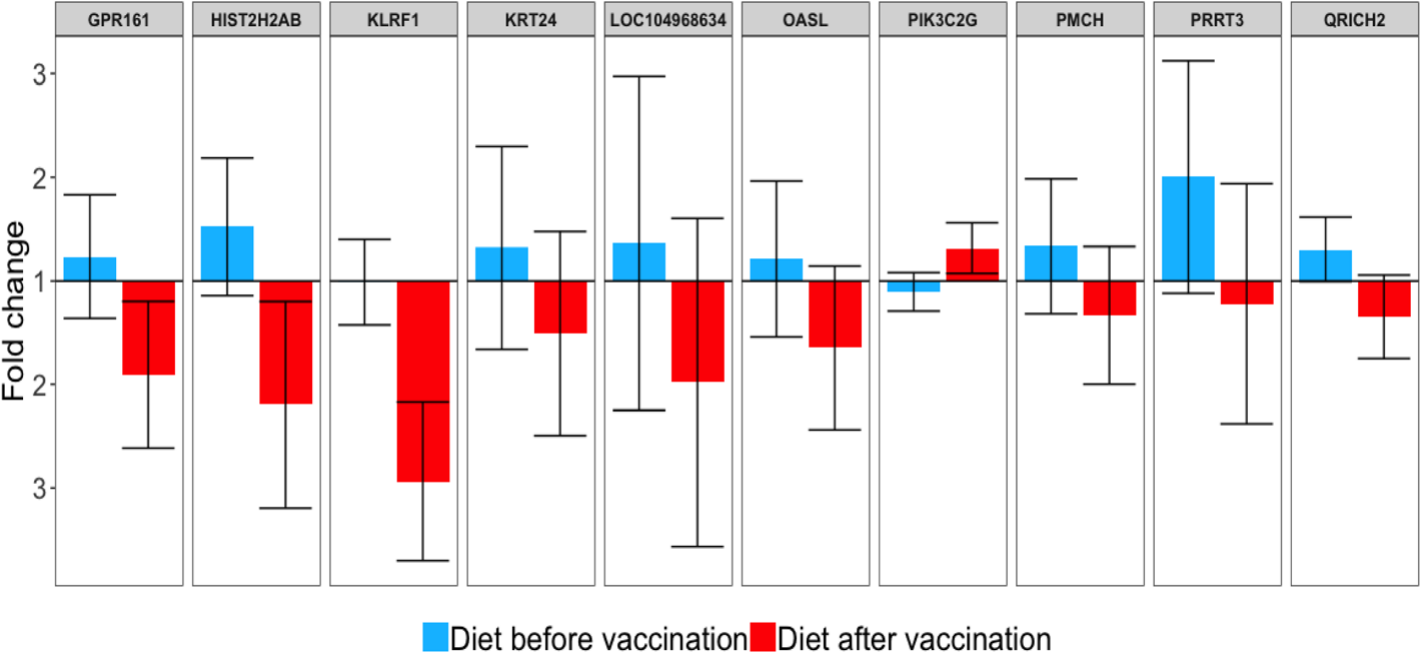
The 10 most differentially expressed genes (*q*-value < 0.05) showing interaction between the effects of diet and vaccination in the blood tissue. Bars represent the fold change (FC) for the effect (*P*-value < 0.05) of diet before (blue) and after (red) vaccination, with error bars representing the 95% confidence interval. Fold change values equal to 1 represent same expression between the diets (control and restricted). Fold change values on the top and bottom halves represent up-regulated DEG in the restricted and control groups, respectively. The name and function of these genes are summarized on Table 4

The effect of the interaction diet-by-time was analyzed based on biologically relevant contrasts. The 10 most significant DEG for the effect of interaction diet-by-weaning and diet-by-vaccination are summarized in Table 4. Of these, the most significant gene for the effect of interaction diet-by-weaning was *USF3* (*q*-value = 1.13E-04) and for the effect of diet-by-vaccination was *LOC104968634* (*q*-value = 6.62E-04). In addition, we found differential expression in genes related to immune system and response to stress, such as *KLRK1,* which stimulates the natural killer (NK) cells [50] and *INO80D* [51], involved in oxidation-reduction activity. *KLRK1* was down-regulated and *INO80D* was up-regulated in the REST after weaning (Fig. 3). In accordance with the results in the muscle tissue for the effect of energy-restricted diet, we hypothesized that the REST triggered a more pronounced stress process than the CRTL during the acute response phase. When we looked at the effect of interaction diet-by-vaccination, we found genes involved in the immune response being down-regulated in the REST after vaccination (Fig. 4), such as *OASL*, which is involved in response to viral infections [52]; *KLRF1*, which is expressed on nearly all NK cells and stimulates their cytotoxicity and cytokine release [50]; and *LOC104968634,* an antimicrobial peptide stimulating NK cells cytoxicity. These findings suggest that the CRTL responded better to the immune challenge which may be due to a better development of the immune system during prenatal phase. Also, these data are in agreement with Moriel et al. [18], who using part of the same data used in this study, showed that 70% of energy restriction during the last 40 days of gestation decreased post-weaning vaccination-induced humoral immunity, inflammatory, and physiological stress responses in calves. Our results support the hypothesis that maternal energy restriction during pregnancy can alter gene expression in the offspring associated with immune response which may reflect on the productive performance of the animal. The complete list of DEG (*q*-value < 0.05) for the effects of diet, time, and interaction diet-by-time is provided in Additional file 4.

**Table 4:** 10 most differentially Expressed Genes^1^ in the blood for biologically relevant contrasts

Gene symbol	Gene name	Biological function	*q*-value
*DEG for effect of interaction diet-by-weaning*			
*USF3*	Upstream transcription factor family member 3	Regulates Major Histocompatibility Complex [82]	1.13E-04
*STARD6*	StAR related lipid transfer domain containing 6	Structure and lipid transport mechanism [83]	3.10E-04
*PHC3*	Polyhomeotic homolog 3		3.17E-04
*BRMS1L*	Breast cancer metastasis-suppressor 1 like	Metastasis suppression [84]	4.87E-04
*MPV17L*	MPV17L mitochondrial inner membrane protein like	Up- or down-regulation of the genes of antioxidant enzymes [85]	4.92E-04
*NBEAL1*	Neurobeachin like 1	Vesicle trafficking, membrane dynamics and receptor signaling [86]	7.31E-04
*C5H12orf4*	Chromosome 5 open reading frame, human C12orf4		7.59E-04
*INO80D*	INO80 complex subunit D	DNA damage responses [51]	8.00E-04
*TIGD3*	Tigger transposable element derived 3		8.94E-04
*KLRK1*	Killer cell lectin like receptor F1	Stimulates natural killer (NK) cells cytoxicity and cytokine release [50]	9.05E-04
*DEG for effect of interaction diet-by-vaccination*			
*LOC104968634*	Antimicrobial peptide NK-lysin-like	Antimicrobial activity [87]	6.62E-04
*GPR161*	G protein-coupled receptor 161	Involved in neural tube development [88]	2.13E-03
*KLRF1*	Killer cell lectin like receptor F1	Stimulates NK cells cytoxicity and cytokine release [50]	2.20E-03
*PMCH*	Pro-melanin concentrating hormone	Feed intake control [89]	2.81E-03
*PIK3C2G*	Phosphatidylinositol-4-phosphate 3-kinase catalytic subunit type 2 gamma	Control cell proliferation [90]	3.15E-03
*KRT24*	Keratin 24		3.64E-03
*PRRT3*	Proline rich transmembrane protein 3	Protein binding [91]	3.74E-03
*QRICH2*	Glutamine rich 2	Cupper regulation [92]	4.18E-03
*HIST2H2AB*	Histone cluster 2 H2A family member B	Play a central role in transcription regulation, DNA repair and DNA replication [93]	4.22E-03
*OASL*	2′-5′-oligoadenylate synthetase like	Response to viral infections [52]	4.73E-03

^1^*P*-value < 0.05 for significant (*q*-value < 0.05) effects of diet-time interaction;

Biological Function, based on cited reference

### Partial correlation network in the muscle and blood

**Fig. 5:**
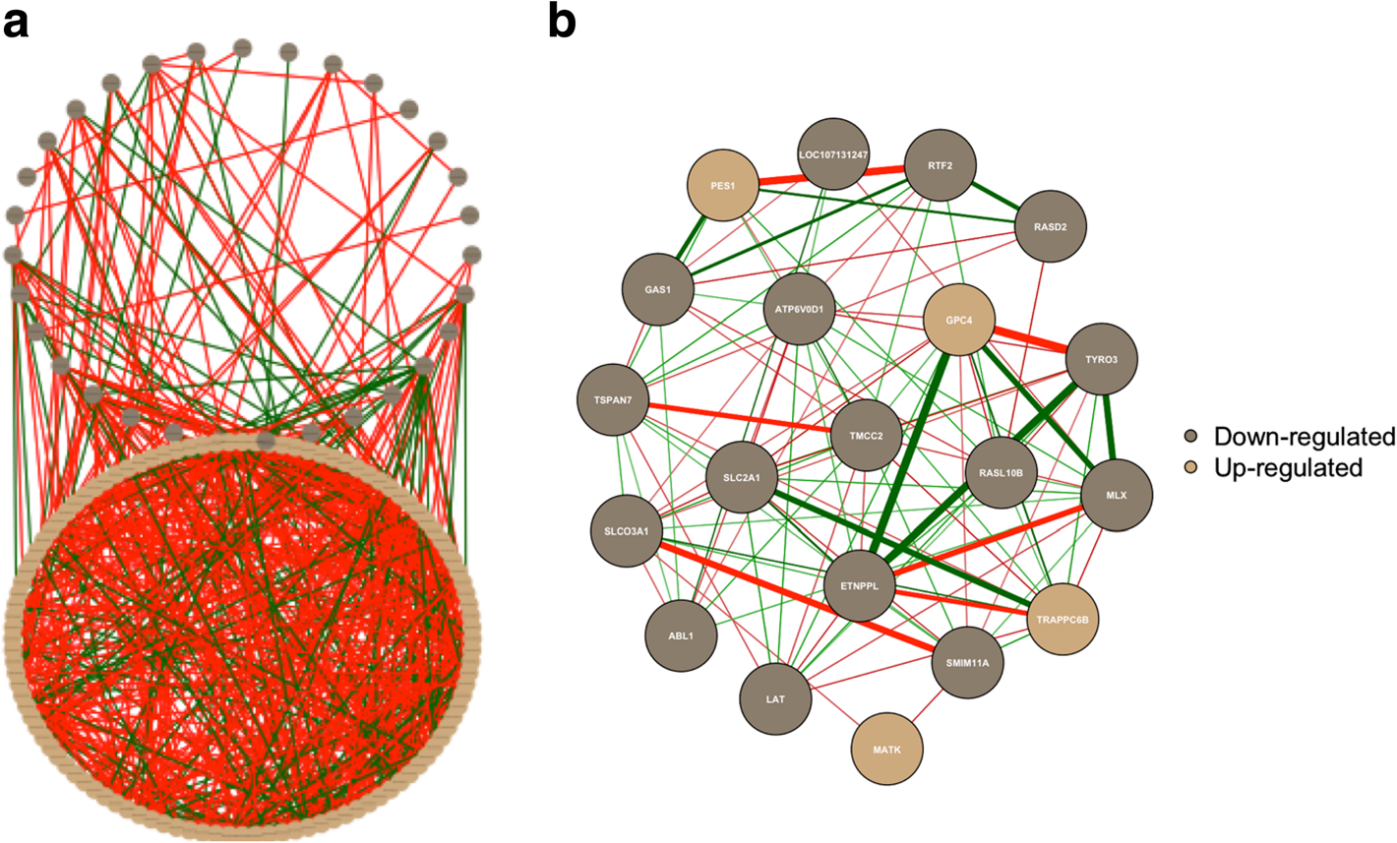
Partial correlation networks for all (**a**) and the 20 (**b**) most differentially expressed genes (DEG; *q*-value < 0.05) for the effect of diet in muscle. Brown and Beige nodes represent down- and up-regulated DEG in the restricted, respectively. Red and green lines represent negative and positive correlation (correlation > |0.8|), respectively

**Fig. 6:**
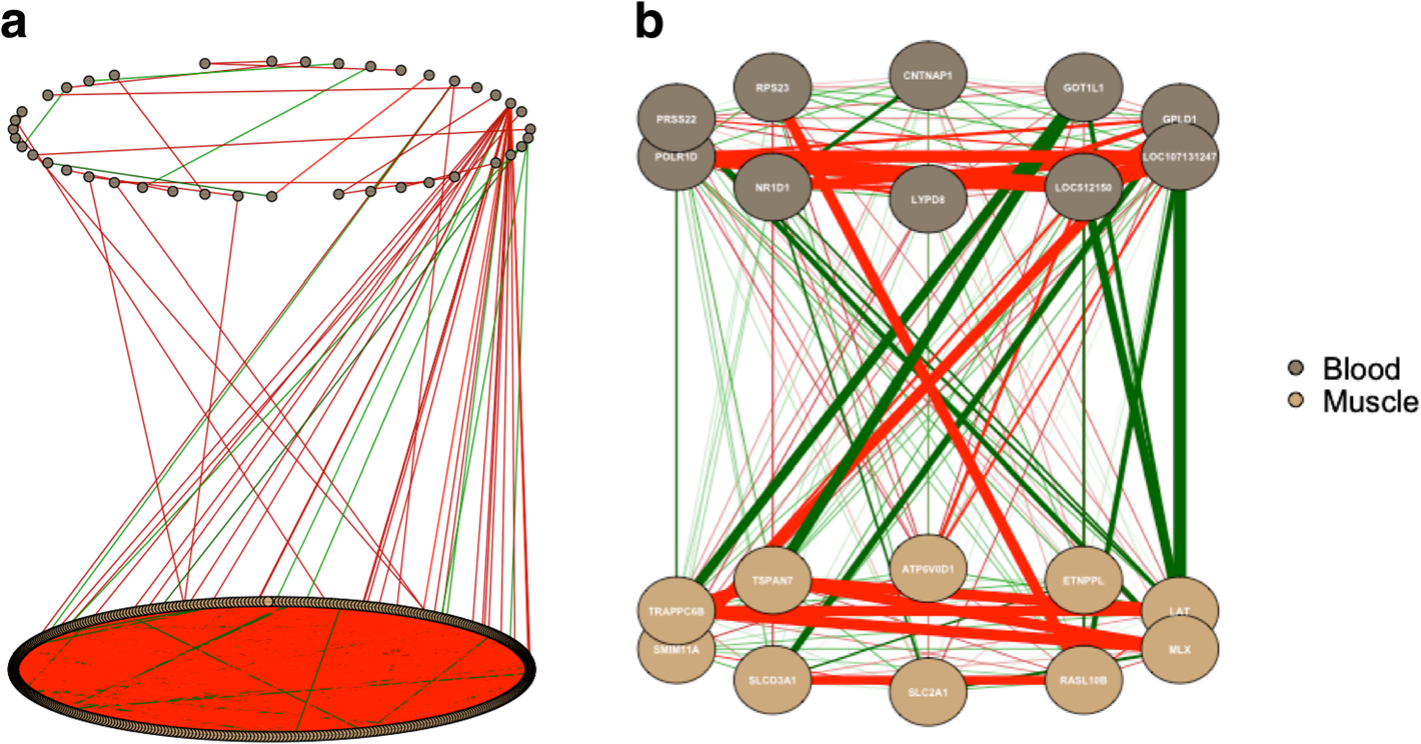
Partial correlation networks for all (**a**) and the 10 most differentially expressed genes (DEG; *q*-value < 0.05) for the effect of diet in muscle associated with the 10 most DEG in blood tissue (**b**). Brown and Beige nodes represent overexpressed DEG in the blood and muscle, respectively. Red and green lines represent negative and positive correlation (correlation > |0.8|), respectively

The partial correlation network including all (Fig. 5a) and the 20 (Fig. 5b) most DEG (*q*-value < 0.05) is depicted in Fig. 5. From the gene network containing all DEG, it seems there are more negative correlations than positive and there is a high correlation between the down- and up-regulated DEG in the muscle. From the network containing the 20 most DEG, the genes with strongest positive correlations were *ETNPPL*, which is involved in the fatty acid metabolism and is being strongly positively correlated with *GPC4* and *RASL10B*, and negative correlated with *MLX* and *TRAPPC6*. The result from the partial correlation network containing all DEG linking muscle and blood (Fig. 6a) showed high negative connectivity between the DEG in the muscle and fewer between muscle and blood. Then, looking at the gene network containing the most 10 DEG in muscle associated with the 10 DEG in blood (Fig. 6b), we can see a higher negative correlation with each tissue but a higher positive correlation between muscle and blood. The gene with higher positive connectivity was GOT1L1, which has been associated with immune response and is been used as cell indicator of stress in cattle semen [53].

Among the DEG in both blood and muscle (*SPAG17*, *VAT1*, *CABLES1*, *SLC20A2*, *ILF3*, *QDPR*, and *LOC107131247*), most of them were down-regulated in the muscle and up-regulated in the blood, except for *SPAG17* and *LOC107131247* which were down-regulated in both. Among the genes down-regulated in the muscle and up-regulated in the blood, some of them are associated with immune system and stress process, i.e. *ILF3* [54], *QDPR* [55], *CABLES1* [56], and *LOC107131247* [57]*. CABLES1* is a glucocorticoid-activated cell cycle regulator, and glucocorticoids are known to be involved in several functions including enhanced response to stress and decreased protein synthesis in skeletal muscle. Therefore, the DEG expressed in both blood and muscle tissue may be playing different roles in each of the tissues.

## Conclusion

This study shows that reducing dietary energy for pregnant cows by 30% during the last 40 days of gestation impacts the transcriptomic profile in the skeletal muscle and blood tissues in the offspring. Maternal energy restriction reduced the expression of genes in the skeletal muscle tissue and increased gene expression in the blood tissue. Specifically, we observed a reduction of genes associated with energy metabolism and muscle development in the skeletal muscle tissue. In the blood tissue, there was a decrease in the expression of genes associated with immune response and stress processes in the REST after weaning and vaccination. Also, we found that the DEG in the muscle and blood showed a connectivity between them, enhancing the importance of the immune system as a target tissue for the effect of maternal energy restriction during late pregnancy. These finding suggest that energy restriction during late pregnancy may trigger a more pronounced stress response in the offspring that may impair the muscle tissue and immune system development.

## Additional files

Additional file 1: Gene set enrichment analysis output for muscle. Excel file with term ontologies from the enrichment analysis. The tabs correspond to each effect of diet and sex in the muscle tissue. The columns represent: (1) GO terms, (2) number of genes associated with the GO term in the reference genome, (3) number of genes associated with the GO term in the list of differentially expressed genes (DEG), (4) number of genes associated with the GO term associated expected in the in the list of DEG, (5) signal representing over-representation (+) or under-representation (−), (6) fold enrichment, and (7) *P*-value. (XLSX 44 kb)

Additional file 2: Differentially expressed genes in muscle. Excel file with all differentially expressed genes (DEG; *q*-value < 0.05). The tabs correspond to each effect of diet, sex and diet-by-sex in the muscle tissue. The columns represent: (1) Ensembl gene ID, (2) HGNC gene symbol, (3) *q*-values (FDR corrected *P*-values), (4) fold change, values between 0 and 1 being down-regulated and values bigger than 1 representing the up-regulated in the REST, and (5) standard errors of the fold change. (XLSX 215 kb)

Additional file 3: Gene set enrichment analysis output for blood. Excel file with term ontologies from the enrichment analysis. The tabs correspond to each effect of diet and time in the blood tissue. The columns represent: (1) GO terms, (2) number of genes associated with the GO term in the reference genome, (3) number of genes associated with the GO term in the list of differentially expressed genes (DEG), (4) number of genes associated with the GO term associated expected in the in the list of DEG, (5) signal representing over-representation (+) or under-representation (−), (6) fold enrichment, and (7) *P*-value. (XLSX 15 kb)

Additional file 4: Differentially expressed genes in the blood. Excel file with all differentially expressed genes (DEG; *q*-value < 0.05). The tabs correspond to each effect of diet, time and diet-by-time in the blood tissue. The columns represent: (1) Ensembl gene ID, (2) HGNC gene symbol, (3) *q*-values (FDR corrected *P*-values), (4) fold change, values between 0 and 1 being down-regulated and values bigger than 1 representing the up-regulated in the REST, and (5) standard errors of the fold change. (XLSX 102 kb)

## Abbreviations

AIC: Akaike information criterion
*ATP6V0D1*: ATPase H+ transporting V0 subunit d1
*CABLES1*: Cdk5 and Abl enzyme substrate 1
CTRL: control group
DEG: differentially expressed genes
DF: degree-of-freedom
DPW: days post weaning
*EIF2S3*: Eukaryotic translation initiation factor 2 subunit gamma
*ETFRF1*: Electron transfer flavoprotein regulatory factor 1
*ETNPPL*: Ethanolamine-phosphate phospholyase
FC: fold change
FDR: false-discovery rate
*FZD5*: Frizzled class receptor 5
*GCGR*: Glucagon receptor
GO: gene ontology
GOT1L1: Glutamic-oxaloacetic transaminase 1 like 1
*GPC4*: Glypican 4
*GTF2A1L*: General transcription factor IIA subunit 1 like
*ICMT*: Isoprenylcysteine carboxyl methyltransferase
*ILF3*: Interleukin enhancer binding factor 3
*INO80D*: INO80 complex subunit D
*KDM6A*: Lysine demethylase 6A
*KLRF1*: Killer cell lectin like receptor F1
*LOC104968634*: Antimicrobial peptide NK-lysin-like
*LOC107131189*: Eukaryotic translation initiation factor 2 subunit 3, Y-linked-like
*LOC107131247*: Multidrug resistance-associated protein 4-like
*LOC512150*: Myeloid-associated differentiation marker-like
*MATK*: Megakaryocyte-associated tyrosine kinase
NK: natural killer
*OASL*: 2′-5′-oligoadenylate synthetase like
*QDPR*: Quinoid dihydropteridine reductase
*RASD2*: RASD family member 2
REST: energy-restricted group
*RIMS1*: Regulating synaptic membrane exocytosis 1
RIN: RNA integrity number
*SLC20A2*: Solute carrier family 20 member 2
*SLCO3A1*: Solute carrier organic anion transporter family member 3A1
*SPAG17*: Sperm associated antigen 17
*SYAP1*: Synapse associated protein 1
*SYT3*: Synaptotagmin 3
*TMCC2*: Transmembrane and coiled-coil domain family 2
TMM: Trimmed Mean of M-values
*USF3*: Upstream transcription factor family member 3
*USP9X*: Ubiquitin specific peptidase 9, X-linked
*VAT1*: Vesicle amine transport 1
